# Recent advances in the role of excitation–inhibition balance in motor recovery post-stroke

**DOI:** 10.12703/r/10-58

**Published:** 2021-06-23

**Authors:** Ioana-Florentina Grigoras, Charlotte J Stagg

**Affiliations:** 1 Wellcome Centre for Integrative Neuroimaging, FMRIB, Nuffield Department of Clinical Neurosciences, University of Oxford; Medical Research Council Brain Network Dynamics Unit, University of Oxford, Oxford, UK

**Keywords:** GABA, stroke, motor recovery, TMS, MRS, NIBS, pharmacological intervention

## Abstract

Stroke affects millions of people worldwide each year, and stroke survivors are often left with motor deficits. Current therapies to improve these functional deficits are limited, making it a priority to better understand the pathophysiology of stroke recovery and find novel adjuvant options. The excitation–inhibition balance undergoes significant changes post-stroke, and the inhibitory neurotransmitter γ-aminobutyric acid (GABA) appears to play an important role in stroke recovery. In this review, we summarise the most recent studies investigating GABAergic inhibition at different stages of stroke. We discuss the proposed role of GABA in counteracting glutamate-mediated excitotoxicity in hyperacute stroke as well as the evidence linking decreased GABAergic inhibition to increased neuronal plasticity in early stroke. Then, we discuss two types of interventions that aim to modulate the excitation–inhibition balance to improve functional outcomes in stroke survivors: non-invasive brain stimulation (NIBS) and pharmacological interventions. Finding the optimal NIBS administration or adjuvant pharmacological therapies would represent an important contribution to the currently scarce therapy options.

## Introduction

Ten million people worldwide have a stroke each year^1^, making stroke a leading cause of both morbidity and mortality^2,3^, with survivors often left with motor impairments that limit their independence^1,4^. Not only is stroke associated with life-changing challenges for the individual and their family^5^ but also it comes with a high economic cost. In 2012, stroke cost the USA alone over $30 billion, a figure predicted to triple by 2030^6,7^.**

Reperfusion therapy has transformed acute stroke care over the last decade^8^, contributing to substantially reduced mortality rates^1^. However, over half of stroke survivors require rehabilitation for residual motor deficits^9,10^. Currently, the gold standard interventions for motor rehabilitation are limited to physical/occupational therapies: patients are given task-specific training^11^, which focuses on gaining new motor skills, constraint-induced movement therapy (CIMT)^12^, or robotic therapy^13,14^. Even after physical therapy, patients may still have motor deficits, deeming the motor recovery incomplete. All current therapies depend on performing repeated movements, limiting their utility in people with severe motor impairments who most require intervention. Therefore, developing novel approaches to enhance the benefits of physiotherapy, particularly for patients with severe residual deficits, is a substantial unmet clinical need. To do this, we need to understand the neural mechanisms of underlying post-stroke motor recovery.

The neural mechanisms of post-stroke recovery have been extensively studied, both in the acute, post-ischaemic period and in the chronic phase. Of these, excitation–inhibition balance appears to be central. Physiologically, both glutamate and γ-aminobutyric acid (GABA), the major excitatory and inhibitory neurotransmitters, are tightly regulated^15^. However, after a stroke, complex changes in GABAergic regulation occur in both stroke survivors and animal models, which are likely central to functional improvement in recovery after stroke^16,17^. Indeed, GABA has been shown to regulate neuroplasticity in the motor cortex in both healthy animal models and humans^18,19^. The modulation of GABAergic signalling is therefore promising for developing novel therapies.

Here, we first discuss how neurotransmitters can be quantified in humans. Then, we review the role of excitation–inhibition balance in the pathophysiology of stroke in both humans and animal models in each stage of stroke recovery. Finally, we describe different putative interventions, which aim to modulate the excitation–inhibition balance in order to improve motor recovery, and discuss what questions remain to be answered.

## GABA quantification in humans

GABA can be quantified in humans using transcranial magnetic stimulation (TMS), magnetic resonance spectroscopy (MRS), and positron emission tomography (PET).

### Transcranial magnetic stimulation

TMS has been used extensively to investigate cortical excitability and intracortical inhibition in stroke patients^20^. Briefly, an electromagnetic coil is positioned on the scalp over the primary motor cortex (M1), which causes a rapidly changing magnetic pulse to induce an electric current in M1 neurons. If the current is large enough, this causes neuronal depolarisation and subsequent muscle contraction, which can be observed as motor-evoked potentials (MEPs) via electromyography (EMG). TMS quantifies overall corticospinal excitability^21^ and can also estimate synaptic activity reasonably specifically by using paired pulses with different interstimulus intervals (ISIs). The two main classes of GABA receptors, GABA_A_ and GABA_B_, can be at least mostly distinguished using quite different stimulus paradigms. GABA_A_ receptor activity can be measured with an ISI of 1–5 ms (short-interval intra-cortical inhibition [SICI])^22^ and GABA_B_ receptor activity with an ISI of 50–200 ms (long-interval intra-cortical inhibition [LICI])^23^.

Even if TMS measurements are widely used to study excitability and inhibition, TMS remains a noisy technique^24^. To get a robust measurement, the mean of a large number of measurements is calculated^25^. The quality of the TMS measurements is also dependent on the experimenter’s proficiency, which cannot be assessed from the data. Finally, acquiring TMS measurements in stroke survivors is difficult, as corticospinal tract damage reduces MEPs. Therefore, TMS studies in stroke patients are biased towards patients with mild and moderate motor impairments.

### Proton magnetic resonance spectroscopy

Proton MRS (^1^H-MRS) allows reliable and non-invasive quantification of neurochemicals *in vivo*^26^. ^1^H-MRS can provide measurements of both glutamate and GABA but with limited spatial and temporal resolution: MRS quantifies the total amount of a neurochemical in a relatively large MRS voxel of interest (8–15 cm^3^), acquired over minutes. Both glutamate and GABA are important brain metabolites; however, establishing whether the MRS-derived metrics relate to metabolism, neurotransmission, or both is not possible.

It is not clear how MRS and TMS metrics relate. Multi-modal TMS and MRS studies found no link between MRS-derived GABA concentrations ([GABA]) and TMS measures of phasic (synaptic) inhibition^27–30^. A relationship has been proposed between [GABA] in M1 and what is believed to be a TMS measure of extrasynaptic, tonic inhibition^27,31^, but this result has not been replicated in recent studies^29,30^.

### Positron emission tomography

PET uses radioactive tracers to measure specific biochemical processes. The radioactive tracers do not interfere with physiological functions, having a relatively short half-life, and can be extremely biochemically specific. While PET does not have good spatial or temporal resolution, it has provided important insights into stroke pathophysiology, such as differentiating between the stroke core and penumbra in humans^32^.

## Excitation–inhibition balance in stroke pathophysiology

### Stages of stroke recovery in humans

A framework has been recently proposed to describe the stroke recovery timeline^33^. In humans, stroke can be classified into four stages: hyper-acute (0–24 hours post-stroke), acute (1–7 days post-stroke), subacute (early subacute 7 days–3 months; late subacute 3–6 months post-stroke), and chronic (more than 6 months post-stroke), each denoted by distinct pathogenic mechanisms. Briefly, hyper-acute stroke is characterised by increased excitability due to glutamate-mediated excitotoxicity, acute and early sub-acute stroke (or early stroke) by reduced intracortical inhibition (hypothesised to support neuronal plasticity) and a significant recovery of motor functions, and chronic stroke by a normalisation of intracortical inhibition and more stable motor performance.

It is unclear how these stages in human recovery map onto recovery stages in animal models. In rodents, the hyper-acute phase is likely to occur in the first few hours after stroke, a timescale broadly similar to that in humans. However, this is dependent on the stroke model being used. In rodents, the post-ischaemic “sensitive period”, putatively the equivalent of early stroke in humans, lasts for the first 4 weeks. This period is characterised by enhanced long-term potentiation, increased cortical excitability, and decreased inhibition along with increased dendritic spine formation and axonal sprouting, creating a pro-plastic environment^34^. Whether this is recapitulated in humans is not yet known, though in humans the rate of motor recovery peaks around 3 months after stroke, whereas this is seen at approximately 4 weeks in rodents^35^, suggesting the periods may have similarities.

### Hyper-acute stroke

No studies have investigated excitation–inhibition balance this early after stroke in humans, likely because of survivors’ complex medical needs at this time. However, we know from animal models and *in vitro* studies that ischaemia leads to neuronal death primarily via glutamate-mediated excitotoxicity^36,37^, which would lead to an increase in the excitation–inhibition ratio. In line with this, an *in vivo* MRS study in rats reported increased glutamate and taurine at 2 hours post-stroke^38^. Indeed, in humans, early mobilisation (within the first 24 hours after the stroke) led to worse outcomes at 3 months^3,39^.

However, a recent *ex vivo* MRS study in rodents demonstrated decreased glutamate in the ipsilesional hemisphere 1 hour after stroke, reaching a minimum after 24 hours. GABA, glycine, and lactate were all significantly increased in the ipsilesional hemisphere during this timeframe, with GABA levels peaking 3 hours after stroke^40^. These results are unexpected; it is possible that different techniques used to perfuse and fix the tissue samples might affect *ex vivo* measurements.

GABA counteracts the excitotoxic activity of glutamate and has therefore been hypothesised to be neuroprotective. Preclinical studies using benzodiazepines, positive allosteric modulators of the GABA_A_ receptor^41^, demonstrated a decrease in ischaemic damage and improved motor rehabilitation in both rodents and non-human primates^42,43^. However, the beneficial effect of benzodiazepines has not been replicated in clinical trials, as reported by a recent Cochrane systematic review. Patients who received benzodiazepines within 12 hours of symptom onset showed no significant difference in recovery compared to placebo^44^.

Benzodiazepines are commonly prescribed to older adults^45,46^, meaning that some patients were taking benzodiazepines before their stroke. Despite promising evidence from the animal literature, patients who were taking benzodiazepines before stroke had a significantly higher mortality and worse functional outcomes 3 months after stroke^47^. However, though the authors tried to eliminate selection bias, this result may be partly biased by the pre-existing comorbidities that led to the need for benzodiazepine treatment.

Taken together, it is likely that human pathophysiology in the hyper-acute phase is more complex than the pathophysiological changes reported in animal stroke literature. Gaining more insight into the pathophysiology of hyper-acute stroke is necessary in order to provide an alternative therapy for patients who cannot receive reperfusion therapy.

### Acute and sub-acute stroke

Most spontaneous recovery occurs in the acute and early sub-acute stages^33,35,48^. The first 3 months post-stroke are likely to be characterised by neurochemical changes that support neuronal plasticity and spontaneous recovery. Therefore, here we will refer to the acute and early sub-acute stages together as “early stroke”.

Early stroke in humans is likely to be characterised by mechanisms and processes similar to those of the peri-ischaemic sensitive period in rodents^15^, but a lack of cross-species tools means that validating this link is difficult. MRS is a potential modality that might allow relatively direct comparison between rodent and human models, though acquisition protocols and tissue preparation may vary considerably between species. One of the clear findings from the animal literature is the increase in extrasynaptic GABA_A_ tonic inhibition in the peri-infarct area^17^. However, *ex vivo* ^1^H-MRS in rodents demonstrated a significant decrease in NAA, GABA, and glutamate and an increase in glutamine, myo-inositol, and lactate in the ipsilesional hemisphere 7 days after stroke^49^, suggesting that the synthesis of both glutamate and GABA from glutamine may be decreased. It is not clear what effect this change in metabolites 7 days after stroke would have on overall excitation–inhibition balance.

In early stroke in humans, TMS studies demonstrate a reduced excitation–inhibition ratio that increases over time. A recent meta-analysis concluded that thresholds were higher in the ipsilesional hemisphere compared to controls, indicating, as expected, a lower excitation–inhibition ratio in the affected hemisphere post-stroke^50^. It is not clear what drives this decreased excitation–inhibition balance: as might be predicted using PET, GABA_A _activity was shown to be increased at 1 month post-stroke, decreasing to normal at 3 months post-stroke^51^. However, TMS metrics show a significant reduction in GABA_A_ activity (SICI) in the ipsilesional hemisphere compared to both the contralesional hemisphere and healthy age-matched controls^50^.

Furthermore, in a recent relatively small study, MRS-derived measurements of [GABA] in ipsilesional M1 were not significantly different from those in healthy age-matched controls, but [GABA] in the contralesional M1 was significantly lower, leading to an overall higher excitation–inhibition ratio^52^. Interpretation of this result is not simple: the authors also reported higher GABA_B_ activity (LICI) in both hemispheres compared with controls, in contradiction to previous studies^53,54^. Compared with controls, there was also an increase in 1 ms SICI, a putative measure of extra-synaptic activity, in the ipsilesional hemisphere at 6 weeks post-stroke, though this was not present at either 2 weeks or 12 weeks post-stroke^52^.

It is difficult to explain why during early stroke patients seem to have both a lower excitation–inhibition ratio and lower intracortical inhibition, especially since there are no significant differences in TMS- or MRS-derived metrics of glutamate activity. Likely, the mechanisms regulating the excitation–inhibition balance are more complex than simply expecting that a change in inhibition would produce a proportional change to the overall excitation–inhibition ratio. Particularly, different aspects of inhibitory signalling (tonic versus phasic inhibition; synaptic GABA_A_ or GABA_B_ receptors) may affect the excitation–inhibition balance in different ways. Pharmacological studies enable modulation of these processes separately, at least to some degree, which may allow researchers to tease apart how changes in either excitation or inhibition affect the overall excitation–inhibition balance.

Conducting research with early stroke patients is difficult, as there are several challenges associated with their care and health status at this point, including difficulties recruiting patients owing to poor recovery, loss of contact because of transfer to a different centre/physiotherapy facility, heterogeneity of comorbidities, medication and recovery, and difficulties in travelling from their home to the research facility, meaning that large samples are often difficult to achieve for individual studies. However, multi-centre studies are hampered by a lack of standardisation in the research methods themselves, for example defining appropriate inclusion criteria when residual functional impairments are not yet clear. In particular, neuroimaging studies in early stroke often rely on clinical imaging, which is not standardised across centres, making multi-centre studies difficult. One way to address some of these challenges is to have a recognised framework of definitions and standards for stroke recovery, such as the one that has been introduced by the Stroke Recovery and Rehabilitation Roundtable taskforce^33^. Adoption of this framework will allow more multi-centre trials to be conducted, ultimately increasing both the quantity and the quality of the data being collected.

### Chronic stroke

Chronic stroke patients are unlikely to improve their motor function after approximately 6 months post-stroke unless given rehabilitation, suggesting that the neurochemical milieu is significantly different in this period compared to in early stroke. However, characterising this has proved difficult: although the majority of studies in humans occur in the chronic phase, there is a paucity of data from animal models regarding excitation–inhibition balance. TMS studies have shown that, as in the early stages of stroke recovery, overall cortical excitability is decreased in the ipsilesional hemisphere compared to both the contralesional hemisphere and healthy age-matched controls^50^. A number of human MRS studies have demonstrated a decrease in [GABA] in the ipsilesional hemisphere at rest in chronic stroke patients^55–57^. However, GABA_A_-mediated inhibition measured using SICI does not differ significantly between the ipsilesional hemisphere and either the contralesional hemisphere or healthy age-matched controls^50^. This pattern contrasts with that seen in early stroke, when GABAergic inhibition is decreased in the ipsilesional hemisphere, but is consistent with the time course for spontaneous functional improvements. Therefore, it is plausible that decreased inhibition in early stroke may play an important role in supporting neuroplasticity and, as inhibition normalises, the potential for spontaneous motor recovery reduces. Recent preliminary evidence supports this role for GABA_A_-mediated inhibition by demonstrating a relationship between lower inhibition and better hand function post-stroke^58^. Improvements in function following rehabilitation interventions at this stage also appear to be related to changes in inhibition. The relative improvement in function after 2 weeks of CIMT was positively correlated with decreases in MRS-derived [GABA] in ipsilesional M1^56^. Moreover, baseline [GABA] predicted subsequent response to a non-invasive brain stimulation (NIBS) intervention^59^.

However, as with all clinical studies, the population studied is heterogeneous and measures are indirect, meaning that drawing firm conclusions from these data can be difficult. In particular, it is likely that rehabilitation interventions modulate excitation–inhibition balance^60–63^; without controlling for this, it is impossible to disambiguate spontaneous changes from those induced by motor training.

Multimodal studies are vital to interpret the potentially conflicting results between modalities. A recent study using both TMS and MRS described no significant differences in TMS-derived measurements of GABA_A_ activity between patients and controls^57^. However, the authors reported a significant reduction in [GABA] in both hemispheres in the same patients. There was no correlation between neurophysiological measurements of GABA_A_ and GABA_B_ receptor inhibition and MRS-derived [GABA], making it difficult to distinguish which GABA compartments are measured with MRS, since they do not relate to the neurotransmitter’s activity at its receptors. This raises an important question for the understanding of brain physiology, which should be addressed by future research: do MRS neurochemical metrics relate more closely to neurotransmitter activity or to metabolism?

One promising avenue for future research highlighted by Mooney and colleagues was the role of the GABA_B_ receptor in the excitation–inhibition balance in chronic stroke. TMS measures of GABA_B_-mediated inhibition have been found to be higher in the ipsilesional hemisphere, and late cortical disinhibition (LCD), a measure of presynaptic GABA_B_ receptors normally present in healthy adults^64^, had an inhibitory effect in the ipsilesional hemisphere in chronic stroke^57^. LCD was the only metric that correlated with clinical impairment and functional scores, possibly suggesting a central role for GABA_B_ signalling.

All of the studies discussed above relate to measures of GABAergic inhibition at rest. However, GABA_A_-mediated inhibition decreases during healthy movement preparation, allowing movement to occur^65^. An abnormal persistence of GABA_A_-mediated inhibition during movement preparation has been reported in chronic stroke patients^66^. It is not clear whether this is a pathological or compensatory change, but a recent study in healthy subjects demonstrated that as subjects learnt a motor task, GABA_A_-mediated inhibition was maintained for longer^67^, possibly suggesting that the maintenance of pre-movement inhibition may be a compensatory phenomenon.

There is no evidence of neurophysiological changes in the contralesional hemisphere and healthy age-matched adults in either early or chronic stroke^50^, suggesting that the pathological mechanisms of stroke are limited to the affected hemisphere. Supporting the presence of a pro-plastic environment in the ipsilesional hemisphere would therefore be more likely to be beneficial for motor recovery than neuromodulation of the contralesional hemisphere.

## Modulating GABAergic inhibition in stroke

Two major classes of interventions have been employed to modulate GABAergic inhibition and promote recovery post-stroke: NIBS and pharmacological agents.

### Non-invasive brain stimulation

NIBS can be used either to provide neurophysiological measurements of cortical excitability and intracortical inhibition, as described above for TMS, or to modulate neuronal activity, usually using repetitive TMS (rTMS) or transcranial electric stimulation in the form of transcranial direct current stimulation (tDCS; see 68 for a full description)^69^.

NIBS aims to promote a pro-plastic environment, and its effect on motor recovery post-stroke has been investigated in numerous studies. This vast body of work is not within the scope of this review. Several recent reviews and meta-analyses have discussed the role of NIBS in motor recovery post-stroke^70^, including the effect of rTMS on motor recovery after stroke^71,72^, the effect of tDCS combined with motor practice on stroke recovery^73,74^, and the role of NIBS on recovery of fine motor functions after stroke^75^. Overall, evidence points to a beneficial effect for NIBS in stroke recovery, especially when associated with physical rehabilitation, but further randomised, sham-controlled studies are needed to address the high heterogeneity of NIBS techniques as well as find the optimal start, duration, and frequency of stimulation administration.

### Pharmacological interventions

Several compounds have recently shown promising results in animal models of stroke but have yet to complete clinical trials.

Recently, S44819,****a selective antagonist of the extrasynaptic GABA_A_ α5 receptor with a good safety profile for human use, was developed^76,77^. Reducing pathologically increased tonic inhibition with S44819 during early stroke led to better motor function in mice^78^, with associated increased neuronal viability, decreased peri-infarct astrogliosis, increased brain capillary density, and higher proliferation of neural precursor cells. Despite this promising evidence from preclinical studies, a recent phase II clinical trial reported no significant difference in overall post-stroke recovery after long-term administration of S44819 in early stroke compared with placebo^79^. However, the modified Rankin Scale, the primary endpoint measure, has low sensitivity, and the absence of measurement on motor-specific scales makes it hard to detect small improvements in motor functions. Additionally, to increase recruitment, this clinical trial included all patients who had suffered a cortical or combined cortical-subcortical ischaemic stroke between 2 and 6 days before inclusion in the study. In animal stroke models, S44819 is shown to improve motor function in a lesion that includes the motor cortex, so it is possible that recruiting only patients with M1 lesions may have shown better outcomes. Additionally, the duration of drug administration for each patient was not taken into account, and no TMS or MRS data were collected to confirm that S44819 administration in stroke patients leads to similar changes in GABAergic inhibition as seen in healthy volunteers. Lastly, it is worth noting that sex hormones influence neurotransmitter release, and the animal stroke study used only male mice. Even if the median age of the patients included in the clinical trial is over 67 years old, which would make the majority of female patients included postmenopausal, a potential difference in efficacy between sexes cannot be excluded. Therefore, it is probably too early to dismiss this approach as a line of potential therapy.

S44819 is not the only GABA-modulating drug to be trialled to normalise the increased tonic inhibition present after stroke. Preclinical stroke studies reported a significant improvement of motor functions in rodents after administration of α-GABA_A_ receptor inverse agonists like L-655,708^17,80–82^ or methyl-6,7-dimethoxy-4-ethyl-beta-carboline-3-carboxylate (DMCM)^83^. Owing to safety issues regarding the need to be dissolved in DMSO, L-655,708 will never be used in humans, but DMCM is yet to be trialled in humans.

Data from animal studies using L-655,708 show that a small GABA receptor occupancy (7–14%) is needed to see a strong effect on post-stroke recovery. Therefore, there is a lot of debate on whether a full antagonist or a negative allosteric modulator (NAM), which would allow for titration of receptor occupancy, might be better to use. Further studies could combine pharmacological interventions with neuroimaging or brain stimulation to test this hypothesis.

A potential role for the δ-GABA_A_ receptors, which are primarily found extrasynaptically, has been proposed in motor stroke recovery after administration of the δ-GABA_A_ receptor-positive allosteric modulator DS2 led to decreases in infarct size and significant motor improvements in rodents^84^. The beneficial role of flavonoids, such as 2'-methoxy-6-methyflavone (2'MeO6MF), on motor recovery after stroke has also been linked to δ-GABA_A_ receptors, as the effect was lost in δ-GABA_A_ receptor knock-out animals^85^.

Zolpidem is a hypnotic and selective non-benzodiazepine GABA-positive allosteric modulator for the α1 subtype of the GABA_A_ receptor^86^. In rodents, two recent studies have shown that low doses of zolpidem administered daily from either day 1 or day 3 after stroke significantly improved motor recovery, though it did not affect the size of the lesion^87,88^. Zolpidem is safe to use in humans and has been used in diseases of consciousness after brain injury^89^. However, whether zolpidem might have a beneficial effect on motor recovery after stroke in humans is yet to be investigated. The hypothesis that zolpidem may have an effect on motor recovery is supported by anecdotal evidence from case studies showing improvements in post-stroke aphasia after zolpidem administration^90^.

However, translating results from animal models to clinical populations has proved to be a significant problem in stroke research. This has been recently addressed by the Stroke Recovery and Rehabilitation Roundtable translational working group. Their guidelines aim to change the design of future preclinical studies in order to align the methods and outcome measures used in animal studies to those of clinical trials^91^. Improving translation can be achieved only by understanding the limitations of animal models of stroke, finding appropriate outcome measurements that can be compared between humans and animals, and designing preclinical and clinical experiments that can be easily compared in terms of dose and time of drug administration.

## Conclusions

In this review, we discussed current understanding of the changes in the excitation–inhibition balance that characterise stroke recovery. While it is apparent that this balance undergoes significant changes during hyperacute, early, and chronic stroke, it is still unclear exactly what molecular and cellular mechanisms are underlying these changes during each stroke stage. GABA seems to play a central role in stroke recovery: increased GABAergic inhibition has been hypothesised to be protective during hyperacute stroke by counteracting glutamate-mediated excitotoxicity, while decreased GABAergic inhibition during early stroke has been proposed as a mechanism to support neuronal plasticity and subsequent improvements in motor functions. However, the role of inhibition in stroke recovery is much more complex. Investigating how changes in GABAergic inhibition affect overall cortical excitability and how they eventually relate to functional changes is necessary in order to better understand stroke pathophysiology.
